# Enhancing Bias Assessment for Complex Term Groups in Language Embedding Models: Quantitative Comparison of Methods

**DOI:** 10.2196/60272

**Published:** 2024-11-12

**Authors:** Magnus Gray, Mariofanna Milanova, Leihong Wu

**Affiliations:** 1 Division of Bioinformatics & Biostatistics, National Center for Toxicological Research, US Food and Drug Administration, Jefferson, AR, United States; 2 Department of Computer Science, Donaghey College of Science, Technology, Engineering, and Mathematics, University of Arkansas at Little Rock, Little Rock, AR, United States

**Keywords:** bias, bias measurement, natural language processing, language models, artificial intelligence, input embeddings, AI, assessment, decision-making, AI-powered tool, NLP, application, AI language models

## Abstract

**Background:**

Artificial intelligence (AI) is rapidly being adopted to build products and aid in the decision-making process across industries. However, AI systems have been shown to exhibit and even amplify biases, causing a growing concern among people worldwide. Thus, investigating methods of measuring and mitigating bias within these AI-powered tools is necessary.

**Objective:**

In natural language processing applications, the word embedding association test (WEAT) is a popular method of measuring bias in input embeddings, a common area of measure bias in AI. However, certain limitations of the WEAT have been identified (ie, their nonrobust measure of bias and their reliance on predefined and limited groups of words or sentences), which may lead to inadequate measurements and evaluations of bias. Thus, this study takes a new approach at modifying this popular measure of bias, with a focus on making it more robust and applicable in other domains.

**Methods:**

In this study, we introduce the SD-WEAT, which is a modified version of the WEAT that uses the SD of multiple permutations of the WEATs to calculate bias in input embeddings. With the SD-WEAT, we evaluated the biases and stability of several language embedding models, including Global Vectors for Word Representation (GloVe), Word2Vec, and bidirectional encoder representations from transformers (BERT).

**Results:**

This method produces results comparable to those of the WEAT, with strong correlations between the methods’ bias scores or effect sizes (*r*=0.786) and *P* values (*r*=0.776), while addressing some of its largest limitations. More specifically, the SD-WEAT is more accessible, as it removes the need to predefine attribute groups, and because the SD-WEAT measures bias over multiple runs rather than one, it reduces the impact of outliers and sample size. Furthermore, the SD-WEAT was found to be more consistent and reliable than its predecessor.

**Conclusions:**

Thus, the SD-WEAT shows promise for robustly measuring bias in the input embeddings fed to AI language models.

## Introduction

### Background

When considering the bias in artificial intelligence (AI) and how it can be mitigated, it is key to understand how to measure the bias of interest in order to properly determine the effectiveness of the mitigation technique. One common area for measuring bias in AI is with regard to the input embeddings of the AI model. Input embeddings are how the training and input data are numerically represented in order to make the data understandable to the model. Word and sentence embeddings are 2 common types of input embeddings, and these are likely to capture societal attitudes and display semantic biases [1]. For example, word embeddings may make biased associations between different genders and certain occupations (ie, nurse and female; doctor and male). Thus, in natural language processing (NLP) applications, such as large language models like ChatGPT and LLaMA, addressing bias in this area is of great importance. Several existing methods for measuring bias in input embeddings include the word embedding association test (WEAT), the Sentence Encoder Association Test (SEAT), and the Embedding Coherence Test [2-4]. The WEAT, for instance, has been rather influential, with its derivative, the SEAT, being used by several studies investigating methods of mitigating bias, including Sent-Debias and Auto-Debias [5,6]. Furthermore, the WEAT has been used to assess the stability of word embedding methods (WEMs) [7].

### WEAT: The Word Embedding Association Test

The WEAT was created in 2017 to assess bias within the semantic representations of words in AI, or word embeddings [2], which represent words as a vector based on the textual context in which the word is found. This metric works by considering 2 sets of target terms (eg, science and art terms) and 2 sets of attribute terms (eg, male and female terms). The null hypothesis is that there is no difference between the sets of target words and their relative similarity to the sets of attribute words. Bias is quantified by computing the probability that a permutation of attribute words would produce the observed difference in sample means and, thus, determining the unlikelihood of the null hypothesis [2].

The WEAT was developed to be a statistical test analogous to the implicit association test (IAT), which asked participants to pair concepts or words that they implicitly associate [8]. A total of 10 WEATs were developed based on the documented human biases highlighted by the IAT. In the first WEAT study, Global Vectors for Word Representation (GloVe) word embeddings were used to numerically represent the selected words for each test and, in turn, compute bias scores (see the Methods section for more information about the WEAT’s method of assessing bias). GloVe is an unsupervised learning algorithm for obtaining vector representations for words [9]. The GloVe model was trained on aggregated word-word cooccurrence statistics from a large English language corpus. The resulting word representations capture meaningful linear substructures, allowing for excellent performance on word analogy, word similarity, and named entity recognition tasks [9].

While the WEAT has become somewhat of a standard measure of bias in input embeddings, there are several limitations of using it to measure an AI language model’s bias. First, the WEAT demands 2 distinct term groups for both targets and attributes, which can be challenging without prior knowledge to segregate, especially for nonbinary terms. For instance, age-related terms might include categories like infants, youth, middle-aged, and seniors, complicating differentiation. The WEAT struggles to consider the nuances across such multiple subgroups, limiting its effectiveness in scenarios where terms don’t neatly divide into binary categories. Second, the current groups of terms could be incomplete, potentially introducing unwanted bias. Finally, the original bias calculation (ie, the effect size) is not that robust, and as such, it may be affected by the size or contents of the target or attribute groups. Thus, there is some room to improve this measure of bias, which leads to the focus of this study.

### SEAT: The Sentence Encoder Association Test

The SEAT is a generalization of the WEAT to phrases and sentences, rather than single or compound words [3]. In more detail, this measure of bias modifies the original WEATs by inserting the words into simple sentence templates such as “This is a[n]<word > .” Furthermore, new tests were created to measure additional race- and sex-related stereotypical biases. In the SEAT study, multiple sentence encoders were evaluated, including those for popular language models, such as ELMo [10], GPT [11], and bidirectional encoder representations from transformers (BERT) [12]. In the SEAT, bias is measured the same as in the WEAT.

With sentence encoders becoming increasingly popular in NLP applications, the SEAT is a useful extension of the WEAT for measuring bias within sentence representations. Based on the SEAT study’s results, sentence embeddings typically display less bias than word embeddings, and more recent sentence encoders (such as those for GPT and BERT) exhibit less bias than previous models (such as GloVe) [3]. However, the SEAT still shares the same major limitations as the WEAT, with the need to have predefined, binary sets of targets and attributes, for instance.

### Applications of WEAT and SEAT

The WEAT [2] has been influential in the investigation and development of techniques for mitigating bias in AI systems, with its sentence-level extension, the SEAT [3], being used to measure and evaluate biases present in sentence presentations before and after applying debiasing techniques. More specifically, the SEAT has been used to evaluate the performance of Sent-Debias and Auto-Debias [5,6]. On the one hand, Sent-Debias [5] is a method of debiasing sentence embeddings, while on the other hand, Auto-Debias [6] is an automatic method of mitigating social biases in pretrained language models that alters the model’s parameters through fine-tuning. Both methods used 6 SEAT benchmarks to measure the bias present in the sentence embeddings for various language models, both before and after their application.

The WEAT has also been used in the study of the stability of WEMs. In 2021, Borah et al [7] developed a metric for stability and evaluated a collection of WEMs, including fastText [13], GloVe [9], and Word2Vec [14], on a collection of downstream tasks, including fairness evaluation. For this task, they used the WEAT’s bias score to assess the stability of the WEMs, noting a relationship between these scores and those of their developed stability metric. Thus, the WEAT can be used to determine the stability of a WEM, which is significant because stability is necessary to produce similar results across multiple experiments.

### Objective

Altogether, with the WEAT’s ability to assess the stability of WEMs and the SEAT’s use in evaluating the performance of recent debiasing techniques such as Sent-Debias and Auto-Debias, it is evident that the WEAT has a large influence in this area of measuring bias in input embeddings. However, certain limitations of the WEAT and SEAT have been identified (ie, their nonrobust measure of bias and their reliance on predefined, binary groups of words or sentences), which may lead to inadequate measurements and evaluations of bias. Thus, this study takes a new approach at modifying this popular measure of bias, with a focus on making it more robust and applicable in other domains. Moreover, we aim to make a more flexible and user-friendly approach to measuring bias in input embeddings, where the user is not required to segregate terms to achieve a reliable bias assessment result.

## Methods

### WEAT Method

The WEAT’s measure of bias, the effect size, is calculated similarly to Cohen *d*; with target sets *X* and *Y* and attribute sets *A* and *B*, the WEAT’s effect size (*d*) is a normalized measure of how separated the 2 distributions of associations between the target and attribute are. In the formula for the effect size (Textbox 1), *s(w,A,B*) measures the association of a target word (*w*) with the attribute words (Textbox 2).

Textbox 1. **Word embedding association test effect size.**

$d=\frac{mean_{x\in X}s\left(x,A,B\right)-mean_{y\in Y}s\left(y,A,B\right)}{std\_dev_{w\epsilon X\cup Y}s\left(w,A,B\right)}$

Textbox 2. **Word embedding association test association measure.**

$s\left(w,A,B\right)=mean_{a\in A}\cos\left(\vec{w},\vec{a}\right)-mean_{b\in B}\cos\left(\vec{w},\vec{b}\right)$

To measure the significance of the associations between targets and attributes and determine the unlikelihood of the null hypothesis, the WEAT defines a 1-sided *P* value test with the test statistic *s*(*X,Y,A,B*). The test statistic (Textbox 3) measures the differential association of the 2 sets of target words with the attribute. Textbox 4 shows how the *P* value is calculated, with (*X_i_,Y_i_*)*_i_* denoting all the partitions of *X*∪*Y* into 2 sets of equal size.

Textbox 3. **The word embedding association test’s test statistic.**

$s\left(X,Y,A,B\right)=\sum_{x\in X}s\left(x,A,B\right)-\sum_{y\in Y}s\left(y,A,B\right)$

Textbox 4. **Word embedding association test *P* value.**

$Pr_{i}\left[s\left(X_{i},Y_{i},A,B\right)>s\left(X,Y,A,B\right)\right]$

### SD-WEAT Method

In this study, we introduce the SD-WEAT, a more robust and balanced method for exploring and assessing bias. Instead of using predefined word sets and using the effect size as the bias measurement score, the words are randomly replaced and the SD of multiple effect sizes is computed. This removes the need to predefine the word groups, allowing for the avoidance of biased groups of words. Furthermore, the results are more robust, as the SD is calculated over multiple runs rather than one. Together, this should allow the SD-WEAT to be a more simplistic and accessible measure of bias in input embeddings.

Multiple experiments were conducted to explore the uses of these modifications to the WEAT. In each experiment, GloVe word embeddings were used to numerically represent the words for each test, following suit from the original WEAT study. More specifically, we used the GloVe model “glove.840B.300d” from the Stanford NLP Group [15], which was trained on 840 billion tokens from Common Crawl data, has a vocab size of 2.2 million, and generates 300-dimension vectors. Moreover, other input embedding methods, including those of BERT [12], Sci-BERT [16], and BioBERT [17], have been evaluated in order to explore the differences of their biases, which may potentially come from their training datasets or techniques.

Due to the significance of the attribute sets in both the WEAT’s null hypothesis and effect size calculation, it was decided to focus 2 experiments around replacing the attribute sets. The primary experiment, hereafter named “SD-WEAT,” used the original 10 benchmark datasets used by the WEAT, but the attribute sets were replaced with random word sets from the combined original attribute sets. The secondary experiment, hereafter named “SD-WEAT-Negative-Control,” used the original target data in the 10 benchmark datasets, but the attribute sets were replaced with random word sets derived from the GloVe dictionary.

In the SD-WEAT, for each of the 10 WEATs, the 2 sets of attribute words were pooled together into one list, and then, 100 new tests were constructed, pulling 4 words from said list to form 2 new attribute sets (of 2 words each). For example, the benchmark WEAT-7, which examines a form of gender bias, contains attribute sets with male (eg, “male,” “man,” “boy,” etc) and female (eg, “female,” “woman,” “girl,” etc) terms. The SD-WEAT forms 100 new tests with attribute sets containing words randomly selected from the combination of these term lists, and as a result, the new attribute sets for one of these tests may resemble the following: (1) “male” and “female”; and (2) “woman” and “boy.” Assuming a normal distribution of attribute set permutations, the SD-WEAT should be capable of assessing bias between the target and attribute concepts in a more robust manner, and this should open the door for assessing bias over multiple attribute groups at once. For instance, in the case of racial bias, the SD-WEAT should be able to assess bias over multiple racial groups such as Asian, Black, or White; the different permutations could be examined at an individual level to further assess bias in this multilevel context. This concept will be further explored in future studies.

**Figure 1.**
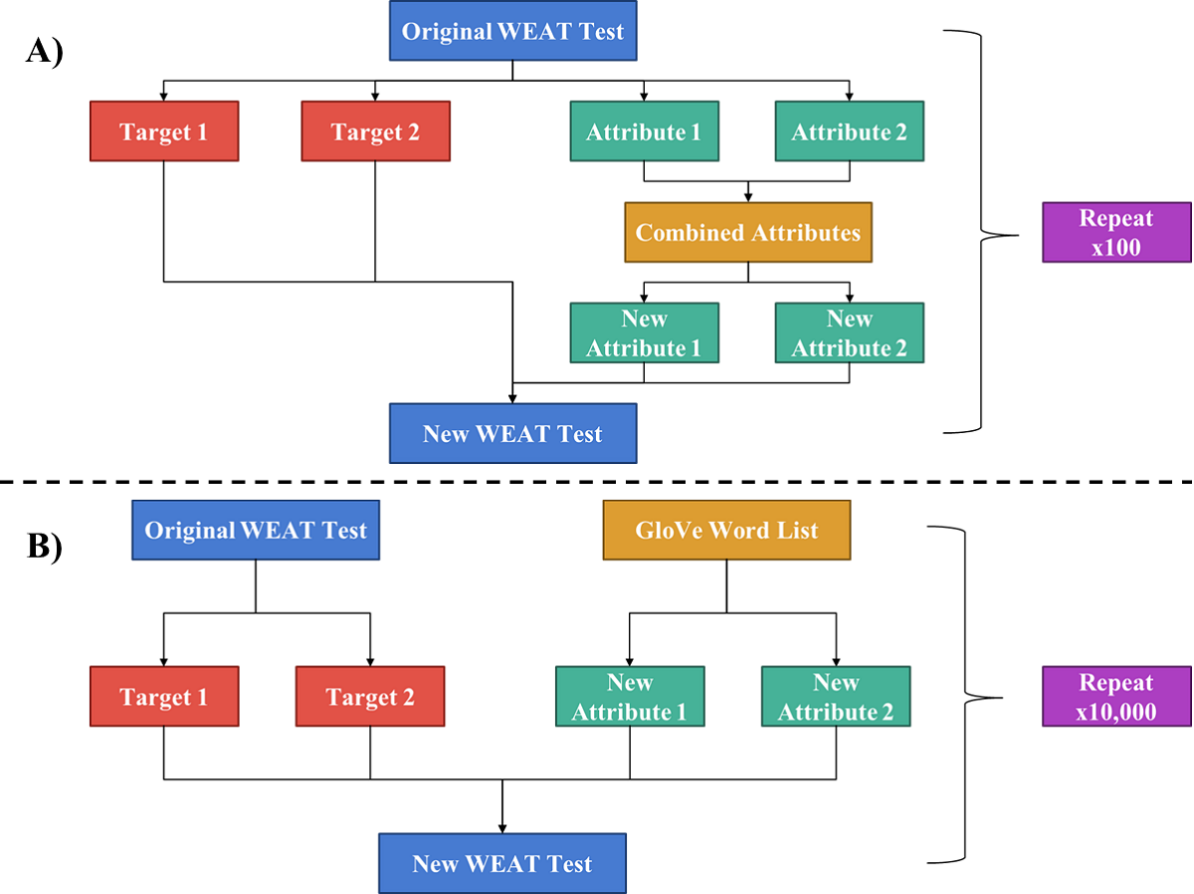
SD-WEAT experiment design. GloVe: Global Vectors for Word Representation. WEAT: word embedding association test.

On the other hand, in the SD-WEAT-Negative-Control, a large list of words was derived from GloVe, and then, 10,000 new tests were constructed, pulling 4 words from said list to form 2 new attribute sets (of 2 words each). Because the GloVe dictionary contains multitudes more words than the original attribute sets, a larger number of tests were constructed than before, allowing for 100 groups of 100 tests to analyze the variance across groups. For each experiment, GloVe word embeddings and a Bag-of-Words model were used to complete each test, and the SD of all the collected effect sizes was computed. Figure 1A and B illustrates the process of creating the new tests for the SD-WEAT and SD-WEAT-Negative-Control, respectively.

Textbox 5 shows how the SD-WEAT quantifies bias, using the effect size (*d*) from the WEAT (see Textbox 1). SD was chosen over other metrics (such as average), because with a normal distribution of attribute permutations, it is expected that the average of all effect sizes is close to zero. The significance of the SD-WEAT results was also calculated. In more detail, *z* scores were calculated for each of the 10 WEAT benchmarks, using Textbox 6. Here, *x* is the SD-WEAT score, while *μ* and *σ* are the average and SD of the SDs for the 100 groups of 100 effect sizes in the SD-WEAT-Negative-Control, respectively. Since the WEAT uses a 1-sided, right-tailed test, *P* values were calculated from the *z* scores with the right-tailed methodology.

Textbox 5. **SD-word embedding association test bias calculation.**

$SD\_WEAT=SD\left(d_{1},d_{2},\ldots d_{100}\right)$

Textbox 6. **SD-word embedding association test significance calculation.**

$\begin{array}{l} z=\frac{x-\mu }{\sigma } \end{array}$

where:

x=SD_WEAT

$\{x{\}}_{\text{control}}=\left(SD(d_{1},d_{2},\ldots ,d_{100}{)}_{1},\ldots ,SD(d_{1},d_{2},\ldots ,d_{100}{)}_{100}\right)$

μ=mean(x_control)

σ=SD(x_control)

### Embedding Method Comparison

To explore the differences of biases between various input embedding methods, several additional methods were evaluated, including BERT, SciBERT, and BioBERT. BERT, which stands for *bidirectional encoder representations from transformers*, is a language model that was pretrained using data from the Toronto Book Corpus and English Wikipedia with the tasks of masked language model and next sentence prediction [12]. Compared to GloVe, which was pretrained using data from Common Crawl and generates context-free representations for each word [9], BERT uses the context on either side of a word to generate that word’s representation. Since the WEATs are only composed of single or compound words rather than sentences, it is expected that the representations for these words will not be influenced by context, and thus, this controls one factor that could potentially contribute to differences in bias between these 2 embedding methods. However, it is expected that differences in bias between GloVe and BERT will emerge based on the differences in their algorithms as well as their training datasets.

SciBERT and BioBERT take inspiration from BERT, using a similar architecture but modifying the model’s training process. On the one hand, SciBERT was pretrained on a large corpus of scientific texts (specifically, 1.14 million papers from Semantic Scholar) rather than the more general texts used for pretraining BERT [16]. SciBERT also uses a new vocabulary based on this scientific corpus. On the other hand, BioBERT continues to pretrain BERT with a large collection of PubMed abstracts (keeping the BERT pretraining datasets), resulting in a model that performs well on biomedical tasks [17]. There is great interest in using these models in regulatory science research efforts, and thus, they were included in this analysis. The BERT, SciBERT, and BioBERT models were each obtained from the Hugging Face repository. In more detail, these 3 models are listed in Hugging Face as “bert-based-cased” [18], “allenai/scibert_scivocab_cased” [19], and “dmis-lab/biobert-v1.1” [20], respectively.

### Stability of Embedding Methods

The WEAT has also been used to assess the stability of WEMs, including fastText, GloVe, and Word2Vec. In more detail, Borah et al [7] trained 3 sets of embeddings for each WEM with a Wikipedia article dataset containing approximately 46 million tokens, changing the seed for each embedding set. The 9 trained embedding sets (3 for each WEM) were then evaluated with the WEAT benchmarks, and the highest and lowest WEAT scores for each benchmark were reported for each of the 3 WEMs. The stability of the WEM is based on the range of WEAT scores; WEMs that produced more similar WEAT scores for each benchmark are more stable than those that produced more different WEAT scores. Based on their results, fastText was found to be the most stable, and Word2Vec was found to be the least stable, with GloVe somewhere in the middle [7]. This aligns with their previous findings, and thus, there is a relationship between WEAT scores and the stability of WEMs.

Our study adopts a similar, yet modified, methodology to assess the stability of the same 3 WEMs, as well as BERT, with both the WEAT and SD-WEAT. In more detail, we began by obtaining the dataset. Without access to the dataset used by the referenced study, we downloaded a Wikipedia dataset (on November 3, 2023) that was uploaded to Hugging Face, specifically the version labeled “20220301.en” [21]. The raw dataset contains over 6 million documents (articles) and 138 million sentences. To conserve computational resources and time, 3 random samples were taken from this dataset, each containing 1% of the total number of documents, or 64,587 to be exact. For the fastText, GloVe, and Word2Vec methods, these 3 sample datasets were processed similarly to those in the referenced study. More specifically, the Wikipedia articles were broken down into sentences using the “punkt” tokenizer from the Natural Language Toolkit (NLTK) package for Python. Then, the sentences were broken down into lists of words, removing stop words and converting all text to lowercase. After processing, each sample dataset contains approximately 1.4 million sentences and 19 million words (tokens). For BERT, which is a sentence embedding method, a different approach had to be taken. The BERT model required a BERT tokenizer, so instead of using a pretrained NLTK tokenizer, a collection of BERT tokenizers was trained using the raw sample datasets. The “bert-base-cased” tokenizer was used to initialize the training, and the vocabulary size was set to 32,000. Three tokenizers were trained per sample dataset, changing the seed for each, resulting in 9 unique BERT tokenizers.

Next, the models were trained. For the fastText and Word2Vec methods, the Gensim package for Python and the “FastText” [22] and “Word2Vec” [23] models were used, and for the GloVe method, the Glove-Python package [24] was used. The parameters for these models were set based on those used in the referenced study, with the vector size set to 300, the context window size set to 5, and the number of epochs set to 5. For each WEM, 3 models were trained per sample dataset, changing the seed parameter for each, resulting in 9 models per WEM. On the other hand, for BERT, the “BertForMaskedLM” model [25] from the Hugging Face package for Python was used. This model uses BERT’s default vector size of 768, with the number of epochs set to 5. In total, 9 BERT models were trained, one for each of the BERT tokenizers that were trained. Since BERT is fundamentally different than the other embedding methods, it is expected that results for these models will differ than those of the WEMs; however, the comparison between the WEAT and SD-WEAT may be useful in the examination of the SD-WEAT’s strengths.

Finally, with 9 models each for fastText, GloVe, Word2Vec, and BERT (or 3 models per each of the 3 sample datasets), the stability of these embedding methods could be evaluated. Each model was assessed with the WEAT and SD-WEAT benchmarks, and the variation within these scores could be used to determine the stability of the method. Furthermore, variations within the scores for a specific benchmark can be noted to analyze the areas that have a greater impact on stability than others.

## Results

### SD-WEAT is Correlated With WEAT in Bias Evaluation

**Figure 2.**
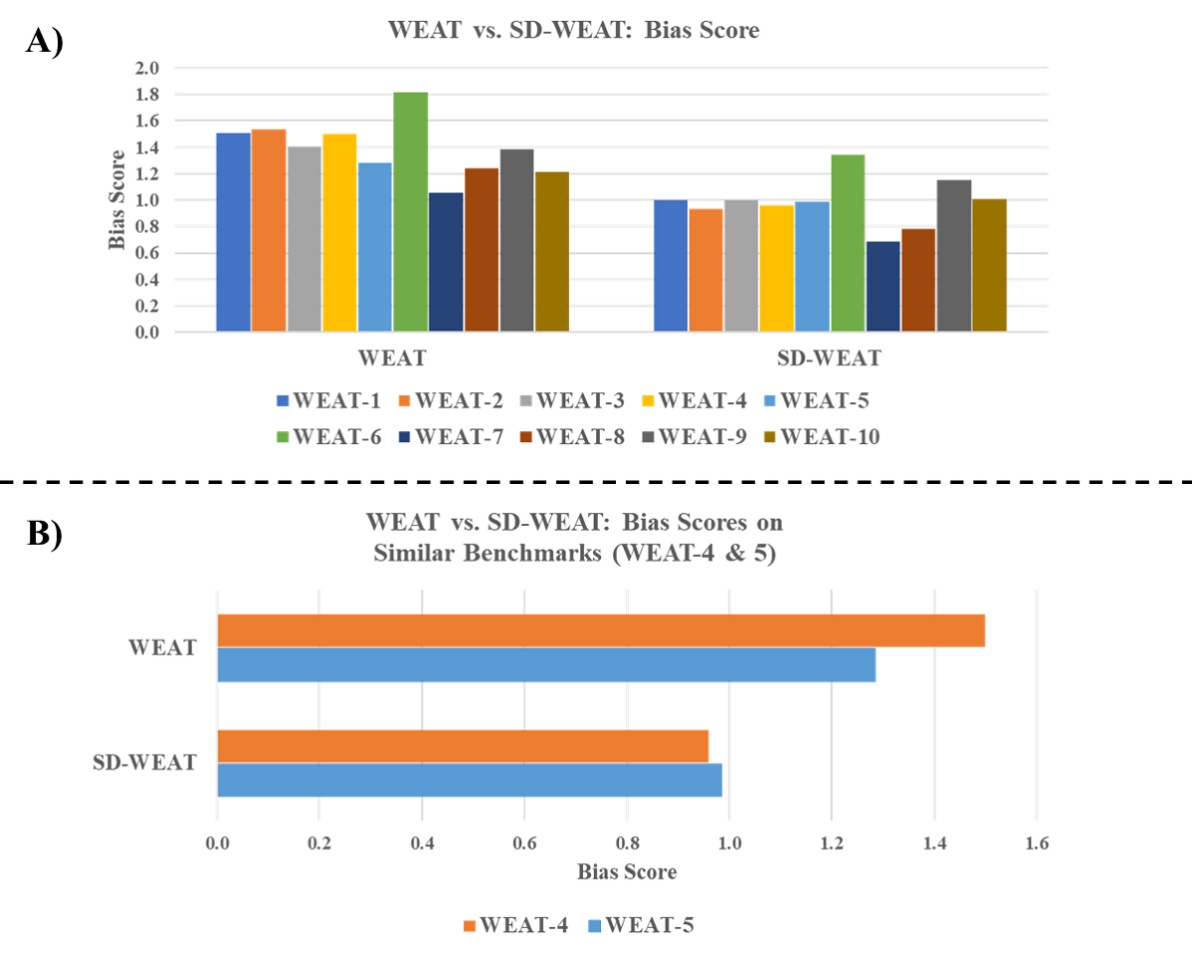
SD-WEAT and WEAT comparison. (A) WEAT versus SD-WEAT bias scores on the 10 WEAT benchmarks. (B) WEAT versus SD-WEAT bias scores on similar benchmarks (WEAT-4 and 5). WEAT: word embedding association test.

Figure 2A illustrates and compares the bias measurement scores for the WEAT and SD-WEAT on the 10 WEAT benchmarks with the GloVe model (see Table 1 for a more detailed breakdown of the results). As shown in Figure 2A, the effect sizes (*r*=0.786) and *P* values (*r*=0.776) for the 10 WEAT benchmarks between 2 approaches were highly correlated. The results demonstrate that the SD-WEAT can effectively measure bias in binary-group attribute terms, providing similar performance as the original WEAT.

**Table 1.** SD-word embedding association test (WEAT) embedding method comparison.

Tests	GloVe^a^		BERT^b^		SciBERT		BioBERT	
	W^c^	SD-W^d^	W	SD-W	W	SD-W	W	SD-W
**WEAT-1**								
Bias score	1.50	1.00	0.08	0.32	−0.01	0.23	0.77	0.42
*P* value	<.001	<.001	.40	.55	.51	.97	.003	.97
**WEAT-2**								
Bias score	1.53	0.93	0.96	1.05	0.43	0.23	0.22	0.29
*P* value	<.001	.02	<.001	.09	.07	.81	.22	.98
**WEAT-3**								
Bias score	1.41	1.00	0.07	0.13	0.32	0.28	0.88	0.73
*P* value	<.001	<.001	.40	>.99	.10	.99	<.001	.28
**WEAT-4**								
Bias score	1.50	0.96	0.42	0.24	0.08	0.36	1.13	0.78
*P* value	<.001	.02	.12	.99	.42	.36	<.001	>.99
**WEAT-5**								
Bias score	1.28	0.98	0.05	0.22	0.31	0.40	.024	0.47
*P* value	<.001	.003	.45	>.99	.20	.01	.25	>.99
**WEAT-6**								
Bias score	1.81	1.35	0.02	0.17	0.40	0.45	0.23	0.27
*P* value	<.001	<.001	.48	>.99	.37	>.99	.33	.97
**WEAT-7**								
Bias score	1.06	0.69	−0.63	0.42	-0.11	0.47	−0.36	0.68
*P* value	.02	>.99	.90	>.99	.58	>.99	.76	.15
**WEAT-8**								
Bias score	1.24	0.78	−0.06	0.15	−0.31	0.51	0.03	0.58
*P* value	.005	.28	.56	>.99	.63	.96	.48	.93
**WEAT-9**								
Bias score	1.38	1.15	1.21	1.05	0.93	0.76	0.01	0.59
*P* value	.004	<.001	.01	.98	.052	.77	.50	<.001
**WEAT-10**								
Bias score	1.21	1.01	0.36	0.31	−0.31	0.85	0.10	0.41
*P* value	.005	.01	.26	.76	.72	.09	.43	>.99

a GloVe: Global Vectors for Word Representation.

b BERT: bidirectional encoder representations from transformers.

c The columns labeled “W” contain the results for the WEAT.

d The columns labeled “SD-W” contain the results for the SD-WEAT.

Additional patterns could be noted between the WEAT and SD-WEAT. Taking a closer look at the WEAT-4 and 5, both shared a similar focus, with the same target sets of European or African names (16 terms each) and different attribute sets formed with some combination of pleasant or unpleasant terms (25 and 8 terms each, respectively). Figure 2B depicts the WEAT and SD-WEAT scores of WEAT-4 and 5, respectively, where the SD-WEAT showed a much closer gap between 2 benchmarks. This suggested that, given the same target terms, the WEAT score was more vulnerable by the size of attribute terms, whereas the SD-WEAT can provide a more consistent effect score for bias assessment.

### SD-WEAT’s Attribute Set Size Did Not Affect Bias Evaluation

In the primary SD-WEAT experiments, the replaced attribute sets consisted of 2 words each, which was the minimum required to measure the effect score. To examine the impact of the attribute size, we also conducted the SD-WEAT experiments with 3- and 5-word attribute sets, keeping other parameters unchanged. Due to the original size of WEAT benchmark datasets, attribute sizes larger than 5 were not tested.

**Figure 3.**
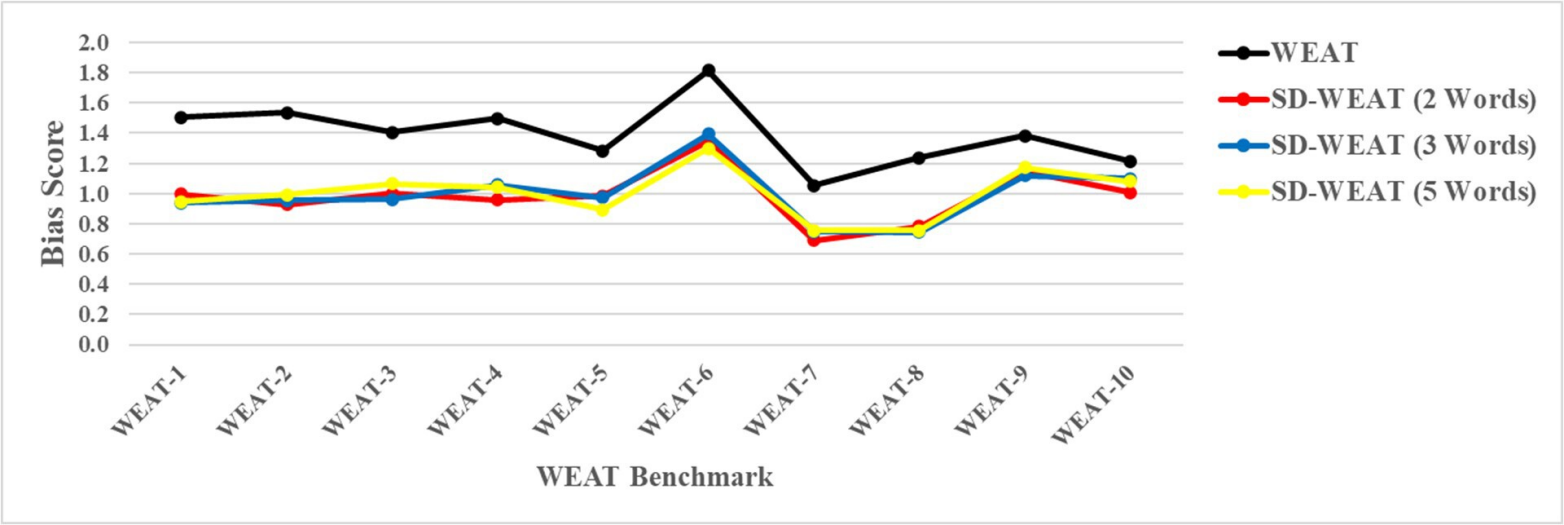
SD-WEAT attribute size analysis (WEAT vs SD-WEAT: bias score). Red, blue, and yellow represent the SD-WEAT with 2, 3, and 5 words, respectively. WEAT: word embedding association test.

The result of this comparative analysis was illustrated in Figure 3. As shown, there was little difference among using 2-, 3-, or 5-word attribute sets. For each attribute size, the correlation between the WEAT’s effect size and the SD-WEAT’s SD is relatively unchanged. Thus, with the SD-WEAT methodology, using any attribute size could provide consistent results.

### Using SD-WEAT to Evaluate Various Embedding Methods

The WEAT and SD-WEAT experiments were further analyzed with BERT, SciBERT, and BioBERT, along with the original embedding method, GloVe, that the WEAT used. Table 1 contains the results. The columns labeled “W” contain the results for the WEAT, while those labeled “SD-W” contain those for the SD-WEAT. Both the bias scores (effect sizes) and *P* values are provided.

Based on the results of this analysis, the 3 BERT input embedding methods typically achieve lower WEAT and SD-WEAT scores compared to the GloVe method, indicating that these methods make less biased associations. Because the BERT embeddings should not be influenced by additional context with the lone words present within the WEAT benchmarks, this shows that the methods’ algorithms or training datasets are likely the cause of their differences in bias. The 3 BERT models have training datasets with more objective language (ie, Wikipedia and scientific articles), which could explain why they produce lower WEAT and SD-WEAT scores than the GloVe method. Furthermore, it can be noted that the various BERT models perform differently than one another. For instance, unlike the other BERT models, BioBERT produces a significant result for WEAT-9, which focuses on the associations between mental and physical diseases and temporary and permanent terms, indicating that the attribute terms used in this specific benchmark have a significantly greater impact than random words. BioBERT was trained on biomedical data, and this could be why it performs so differently for this biomedical task.

### Using SD-WEAT to Assess the Stability of Embedding Methods

The stability of fastText, GloVe, Word2Vec, and BERT was evaluated using the WEAT and SD-WEAT. Multimedia Appendix 1 shows the variability in the WEAT and SD-WEAT scores obtained for each WEAT benchmark over 9 training iterations per embedding method. The subfigures in the left column are box plots showing the spread of the scores, while the subfigures in the right column are bar charts showing the SD of these scores. At a glance, the box plots show that there is less variability in the SD-WEAT scores than the WEAT scores for each benchmark, confirmed by the lower SDs in the bar charts. Furthermore, based on the sizes of the box plots, some embedding methods appear more stable than others. For instance, fastText appears to have less variability in the WEAT and SD-WEAT scores than GloVe, Word2Vec, and BERT, indicating that this method may be the most stable of the ones evaluated.

**Figure 4.**
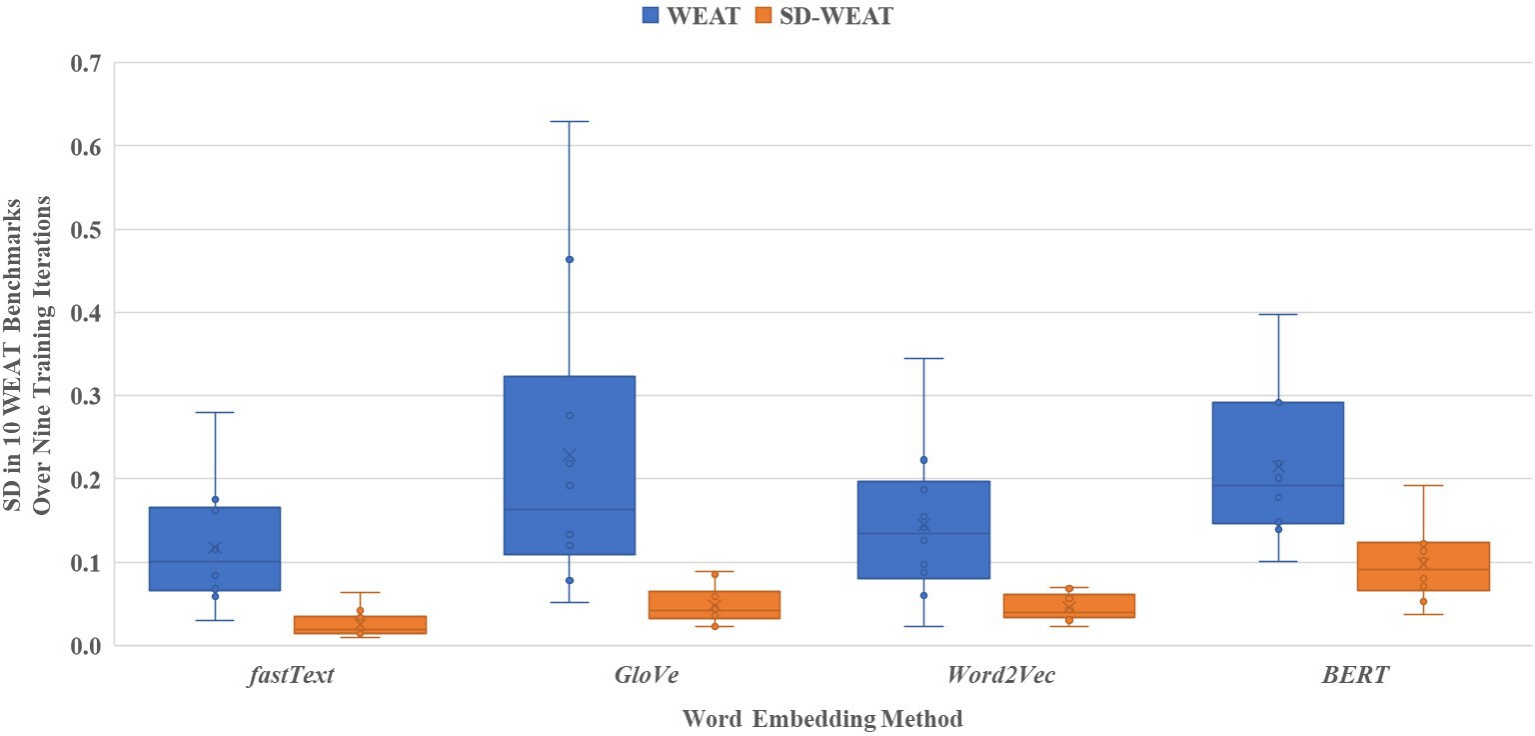
Stability of word embedding methods: BERT: bidirectional encoder representations from transformers; GloVe: Global Vectors for Word Representation; WEAT versus SD-WEAT. WEAT: word embedding association test.

Figure 4 compares the stability of each embedding method based on the SD of the WEAT and SD-WEAT scores over the 10 WEAT benchmarks. Recall that for each embedding method, 9 models were trained. Thus, the SD of the WEAT and SD-WEAT scores was calculated for each set of 9 models for each WEAT benchmark. Since SD is a measure of variation, lower values can be more stable. Again, fastText appears to be the most stable embedding method based on the WEAT and SD-WEAT scores. Furthermore, based on the results, there is less variability in the SD-WEAT scores than the WEAT scores.

Overall, these findings are somewhat different than those of the referenced study, which found fastText to be the most stable and Word2Vec to be the least stable out of the 3 WEMs based on WEAT scores. In our case, fastText was indeed the most stable for the WEAT, but GloVe produced the most variable results. This difference may be a result of the difference in dataset size or contents, as we opted to use 3 smaller random samples than one larger one. This study also analyzed the stability of BERT, a sentence embedding method. However, based on the results, this method produces more variable WEAT and SD-WEAT scores than other methods, which may be a result of the fundamental differences of this model compared to the others. Moreover, some of this instability may be due to the small training sample, as the original BERT model was trained on much more data. Nonetheless, we found that all the models showed less variability with regard to the SD-WEAT than the WEAT. This shows a major strength of the SD-WEAT; this measure of bias is much more consistent and reliable than its predecessor.

## Discussion

### Overview

In this paper, we explored the methodology of measuring bias in the input embeddings of language embedding models, developing a novel approach, called SD-WEAT, for enhancing bias assessment for complex term groups. This method addresses several limitations of its predecessor, the WEAT, resulting in a more robust and consistent measure of bias. Furthermore, with the SD-WEAT, it is now possible to assess bias over multilevel attribute groups, such as age, race, region, etc, in addition to binary attribute groups.

### Future Directions and Limitations

In the future, new benchmarks can be established to measure bias among demographic groups or topics and a full list of attribute terms without segregation. For instance, a benchmark can be developed with target sets comprised of sex-linked medical terms and an attribute set of gender terms in order to estimate the level of bias between sex and medical conditions. In addition, by analyzing the individual trials that produced the highest bias effect, the medical terms that have the greatest association with one sex over the other can be identified. These applications may provide new insight into how these language embedding models can be applied into health care and regulatory science fields.

One limitation of the SD-WEAT is the increased computation resources due to having to generate and execute multiple runs to calculate the final bias effect score. However, the impact is rather minimal, only needing to examine the embedding method’s biases once or periodically.

### Advantages of SD-WEAT

The SD-WEAT not only enables the bias assessment over multilevel group terms but also enhances the bias assessment for binary group terms by avoiding unnecessary groupings of words. In some cases, it may be difficult to determine whether an intermediate word should belong to a certain grouping. Moreover, the SD-WEAT more robustly measures bias through the utilization of the SD of multiple effect sizes, and it has been found to be a more consistent and reliable measure of bias than its predecessor. As such, the SD-WEAT is a more robust and user-friendly measure of bias in input embeddings for AI language models.

### Conclusions

To conclude, we introduced the SD-WEAT, a novel algorithm based on the WEAT, to enhance the bias assessment for complex term groups in language embedding models. In the future, the SD-WEAT could be applied in a regulatory science application and provide new insights to measure biases of common embedding models with regard to multilevel attribute groups, such as age, race, and region.

## Supplementary material

10.2196/60272 Multimedia Appendix 1 Variability in word embedding association test (WEAT) and SD-WEAT scores for the 10 WEAT benchmarks over 9 training iterations.

## Abbreviations

AI: artificial intelligence
BERT: bidirectional encoder representations from transformers
GloVe: Global Vectors for Word Representation
IAT: implicit association test
NLP: natural language processing
NLTK: Natural Language Toolkit
SEAT: sentence encoder association test
WEAT: word embedding association test
WEM: word embedding method
