# Supplementary figures and images for: Enhancing Bias Assessment for Complex Term Groups in Language Embedding Models: Quantitative Comparison of Methods
Source: JMIR Med Inform. 2024 Nov 12;12:e60272. doi: 10.2196/60272 (PMC11611796; doi:10.2196/60272)

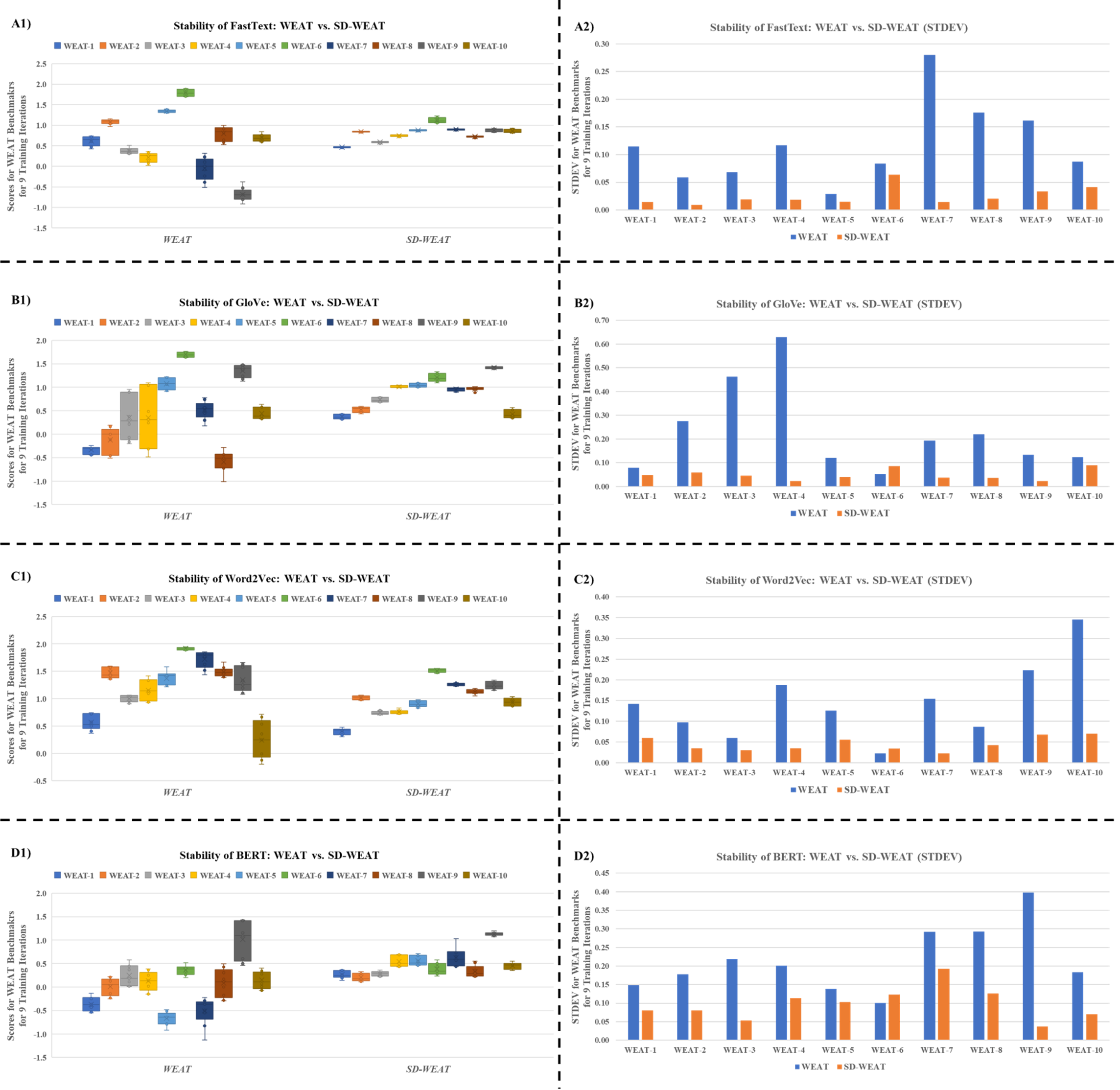

Supplement: Multimedia Appendix 1 [file medinform-v12-e60272-s001.png]
